# Discovery of Small-Molecule PD-L1 Inhibitors via Virtual Screening and Their Immune-Mediated Anti-Tumor Effects

**DOI:** 10.3390/ph18081209

**Published:** 2025-08-15

**Authors:** Chunlai Feng, Yingying Ge, Siqi Wang, Mengru Li, Qiying Chen, Hangyu Dong, Mengjie Rui

**Affiliations:** 1 School of Pharmacy, Jiangsu University, 301 Xuefu Road, Zhenjiang 212013, China; 2212215008@stmail.ujs.edu.cn (Y.G.); swang54@qub.ac.uk (S.W.); 2212415016@stmail.ujs.edu.cn (M.L.); 2232215001@stmail.ujs.edu.cn (Q.C.); 2222415042@stmail.ujs.edu.cn (H.D.); 2 NHC Key Laboratory of Diagnosis and Therapy of Gastrointestinal Tumor, Gansu Provincial Hospital, Lanzhou 730000, China

**Keywords:** PD-1/PD-L1 checkpoint, cancer immunotherapy, virtual screening, pharmacophore modeling, molecular docking, PD-L1 inhibitor

## Abstract

**Background/Objectives**: Monoclonal antibodies targeting the PD-1/PD-L1 immune checkpoint have achieved clinical success but face drawbacks such as poor oral bioavailability, limited tumor penetration, and immune-related adverse events. Small-molecule inhibitors present a promising alternative that may overcome these challenges. **Methods**: Here, an integrated computational framework combining ligand-based pharmacophore modeling and structure-based molecular docking was utilized to screen a comprehensive library consisting of traditional Chinese medicine-derived compounds and clinically approved drugs. The binding affinity between identified candidate compounds and PD-L1 was quantitatively assessed using bio-layer interferometry (BLI). In vitro cytotoxicity assays were conducted on A549 human lung carcinoma and LLC mouse lung carcinoma cell lines. In vivo antitumor efficacy was evaluated in LLC tumor-bearing mice through measurement of tumor growth inhibition, serum cytokine levels (IFN-γ and IL-4) by ELISA, and expression levels of IFN-γ and granzyme B (GZMB) within tumor tissues via immunohistochemistry. **Results**: In vitro, anidulafungin exhibited anti-tumor effects against both human lung cancer A549 cells and mouse Lewis lung carcinoma (LLC) tumor cells, with IC_50_ values of 170.6 µg/mL and 160.9 µg/mL, respectively. The BLI analysis revealed a dissociation constant (K_D_) of 76.9 μM, indicating a high affinity of anidulafungin for PD-L1. In vivo, anidulafungin significantly increased serum levels of IFN-γ and IL-4 in tumor-bearing mice and elevated expression of IFN-γ and granzyme B (GZMB) in tumor tissues, confirming its immune-mediated anti-tumor effects. **Conclusions**: Anidulafungin represents a promising small-molecule PD-L1 inhibitor, demonstrating significant anti-tumor potential via immune activation and highlighting the feasibility of repurposing approved drugs for cancer immunotherapy.

## 1. Introduction

Immune checkpoint molecules play an important role in regulating the immune system and maintaining self-tolerance [1]. Programmed cell death protein 1 (PD-1) and its ligand, programmed death ligand 1 (PD-L1), are among the most extensively studied immune checkpoints [2]. Under normal physiological conditions, PD-L1 is expressed on the surface of immune system cells and interacts with its receptor PD-1, which is a cell surface receptor mainly expressed on activated T cells [3,4]. This interaction inhibits T cell activation and serves as a crucial negative regulator of the immune response [5]. During tumorigenesis, malignant cells often upregulate PD-L1 expression due to the influence of the tumor microenvironment [6]. This overexpression facilitates the binding of PD-L1 to PD-1 on T cells, thereby suppressing T cell activation and inducing T cell apoptosis [7,8,9,10,11]. As a result, tumor immune evasion is enhanced. The overexpression of PD-L1 has been widely associated with poor prognosis across various cancers [12]. Consequently, targeting and inhibiting PD-L1 expression on tumor cells has emerged as a promising strategy in cancer immunotherapy.

Currently, the only approved inhibitors targeting PD-1 or PD-L1 are monoclonal antibodies. While these antibodies have demonstrated significant clinical efficacy [13], they come with certain limitations, such as poor oral bioavailability, limited tumor penetration, and the risk of immune-related adverse events due to systemic immune activation [14,15]. Small molecules, in contrast, offer several potential advantages, including oral administration, enhanced tissue penetration, and the potential to overcome existing therapeutic limitations [16,17,18]. While no small-molecule inhibitors have reached the market yet, significant progress has been made in preclinical and clinical research, with 15 candidates currently in clinical trials as of 2024 [19]. Small-molecule inhibitors target the PD-1/PD-L1 interaction through unique structural designs. For instance, biphenyl-based compounds (e.g., BMS-202 and BMS-1058) induce PD-L1 dimerization, blocking its binding to PD-1 and promoting PD-L1 internalization and degradation [20]. This strongly emphasizes the potential of small-molecule inhibitors targeting PD-1/PD-L1 [21], offering synergistic effects when combined with existing treatments.

Recent advancements in Computer-Assisted Drug Design (CADD) [22] have markedly enhanced the efficiency of small-molecule drug discovery. In particular, pharmacophore models and molecular docking are integral tools in this process, facilitating the identification of novel inhibitors by predicting molecular interactions and optimizing lead compounds. Pharmacophore modeling can be derived from known active ligands (ligand-based) or target binding sites (structure-based), adapting to diverse scenarios with limited structural data. It also enhances efficiency in drug discovery by filtering compounds based on predefined pharmacophoric features. Additionally, molecular docking provides atomic-resolution insights into ligand-target interactions, while modern scoring functions that integrate machine learning further enhance the reliability of affinity predictions. These tools are particularly valuable for challenging targets like protein–protein interactions (e.g., PD-1/PD-L1), where traditional screening often fails to address complex binding landscapes.

In this study, we combined ligand-based pharmacophore modeling and structure-based docking to discover new small-molecule PD-L1 inhibitors. We first built a pharmacophore model based on known PD-L1 inhibitors and used it to screen a library of natural and drug molecules. Top hits were further refined by successive rounds of molecular docking, including focused docking on key interfacial “hotspot” residues of PD-L1. We then evaluated the leading candidates in vitro for PD-L1 binding and tumor cell growth inhibition and in vivo for immune-mediated anti-tumor efficacy in a mouse model.

## 2. Results and Discussion

### 2.1. PD-L1 Ligand-Based Pharmacophore Model Generation and Validation

**Figure 1:**
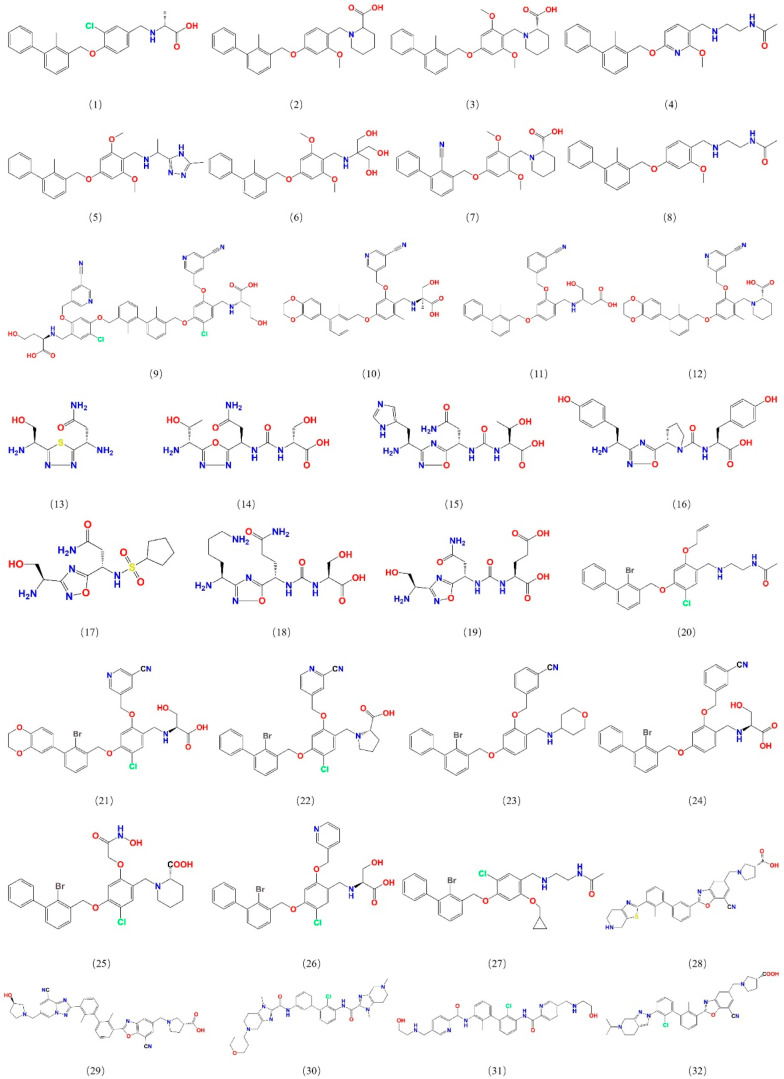
Structures of 32 small molecules for the training dataset.

To construct a pharmacophore model for small-molecule PD-L1 inhibitors, the dataset containing small molecules with PD-L1 inhibitory function was assembled from PubMed and the China National Intellectual Property Administration (CNIPA). These compounds include various chemotypes (biphenyl derivatives, heteroaryl biphenyls, thiazoles, benzyl phenyl ethers, imidazo[4,5-c]pyridines, etc.) with IC_50_ values ranging from 10^−4^ to 10^2^ nM, ensuring a broad activity range and structural diversity. From this collection, 32 potent inhibitors were selected as a training set for pharmacophore development (Figure 1), and an additional 30 compounds were set aside as a test set for model validation. All selected training-set compounds are reported to target the same PD-L1 surface pocket—the PD-1 binding interface—thereby justifying the development of a single, unified pharmacophore model for this site.

**Figure 2:**
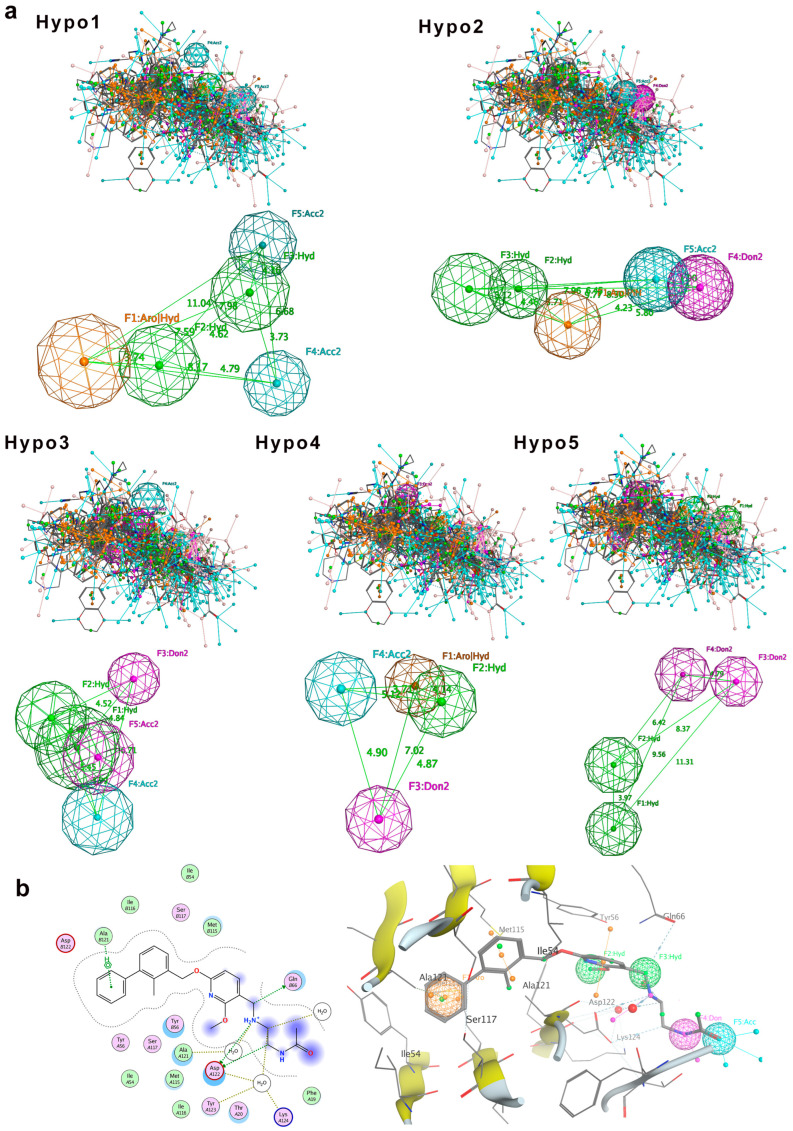
Pharmacophore model generation. (**a**) Five molecular superimposition results and corresponding pharmacophore models. Pharmacophore features are color-coded: blue for hydrogen bond acceptor, purple for hydrogen bond donor, green for hydrophobic features, and orange for aromatic ring features. (**b**) Detailed interaction between PD-L1 and the inhibitor BMS-202, highlighting pharmacophore features of Hypo2: F1 (aromatic ring, R), F2 and F3 (hydrophobic features, H), F4 (hydrogen bond donor, D), and F5 (hydrogen bond acceptor, A).

Pharmacophoric features were superimposed by Molecular Operating Environment (MOE) [23]. The superimposed pharmacophoric features include hydrogen bond acceptor (A), hydrogen bond donor (D), hydrophobic feature (H), and ring aromatic feature (R). After molecular superimposition, five pharmacophore models (Hypo1 to Hypo5) were generated for the training set compounds (Figure 2). Among them, three pharmacophore models (Hypo1, Hypo2, and Hypo3) each contain five pharmacophore features, while the other two models (Hypo4 and Hypo5) contain four features each (Figure 2a).

To evaluate the validity of the pharmacophore models, we first tested the 32 training set compounds. The match rate was calculated as the proportion of compounds that satisfied all pharmacophore features relative to the total number of training dataset compounds. The result indicated that Hypo1, Hypo2, and Hypo4 had all 32 compounds meeting all pharmacophore features, achieving a match rate of 100%. Therefore, Hypo1, Hypo2, and Hypo4 can comprehensively represent the pharmacophore features of the compounds. Notably, these three pharmacophore models incorporate both aromatic ring (R) and hydrophobic (H) features, highlighting that the ring aromatic feature and hydrophobic feature may be the main features of the pharmacophore models. The pharmacophore features of Hypo3 and Hypo5 did not include the ring aromatic feature (R), with match rates of 96.88% and 93.75%, respectively.

Next, we challenged the models with the independent 30-compound test set. The structures of the 30 test-set compounds are provided in Figure S1 (Supplementary Information). Consistently, Hypo1, Hypo2, and Hypo3 identified > 90% of the test set molecules, with Hypo2 performing best (96.7% match rate). Hypo4 and Hypo5 were less effective (73–80% matches). Table 1 summarizes the test set validation results. Given its broad coverage and highest match rate, Hypo2 was chosen as the optimal pharmacophore model for subsequent virtual screening.

To further assess Hypo2’s relevance, we compared it against known PD-L1 inhibitor binding modes. We examined the crystal structures of five prototypical small-molecule PD-L1 inhibitors in complex with PD-L1 (BMS-8, BMS-202, BMS-200, BMS-37, and BMS-1001; PDB IDs 5J8O, 5J89, 5N2F, 5N2D, and 5NIU). Remarkably, Hypo2 perfectly aligned with the crystal pose of BMS-202, a known PD-L1 dimerizer. BMS-202’s aromatic core, hydrophobic substituents, and key hydrogen bonding moieties precisely mapped onto Hypo2’s pharmacophoric features, whereas other inhibitors exhibited partial fits. Visualization of BMS-202 within the PD-L1 binding pocket (Figure 2b) confirmed that its biphenyl core and critical substituents engage PD-L1 in a manner fully consistent with Hypo2’s predicted interactions. Collectively, these results validated Hypo2 as a robust and predictive pharmacophore model [24], suitable for guiding the identification of novel PD-L1 inhibitors.

### 2.2. Virtual Screening with Pharmacophore Model

Using Hypo2 as a 3D query, we screened a compound dataset of 3906 molecules assembled from diverse sources. The dataset comprised two main subsets: (1) 1876 small molecules derived from 90 immunomodulatory and anti-tumor traditional Chinese medicines (TCMs), acquired from the Traditional Chinese Medicine Systems Pharmacology (TCMSP) and PubChem databases, and (2) 2030 approved small-molecule drugs from the Topscience Database (https://www.targetmol.cn/topscience-database, accessed on 22 June 2024). By including both natural products and existing drugs, we aimed to find either novel chemotypes or repurpose known bioactives. Each compound was computationally aligned to Hypo2, and those that matched all five features were selected as hits. The pharmacophore filter yielded 460 compounds (out of 3906) that satisfied Hypo2 completely. These hits were carried forward into the docking stage of the virtual screening.

### 2.3. Binding Site Analysis and Hotspot Identification

To guide the molecular docking and prioritize compounds that could block PD-1/PD-L1 binding, the binding interfaces of PD-1 with PD-L1, with the antibody durvalumab, and with known small molecule inhibitors were analyzed. The key hotspot residues on the PD-L1 binding site were important for binding these partners, so our goal was to identify these hotspots and focus on compounds engaging those same residues.

#### 2.3.1. PD-1/PD-L1 Interface

**Figure 3:**
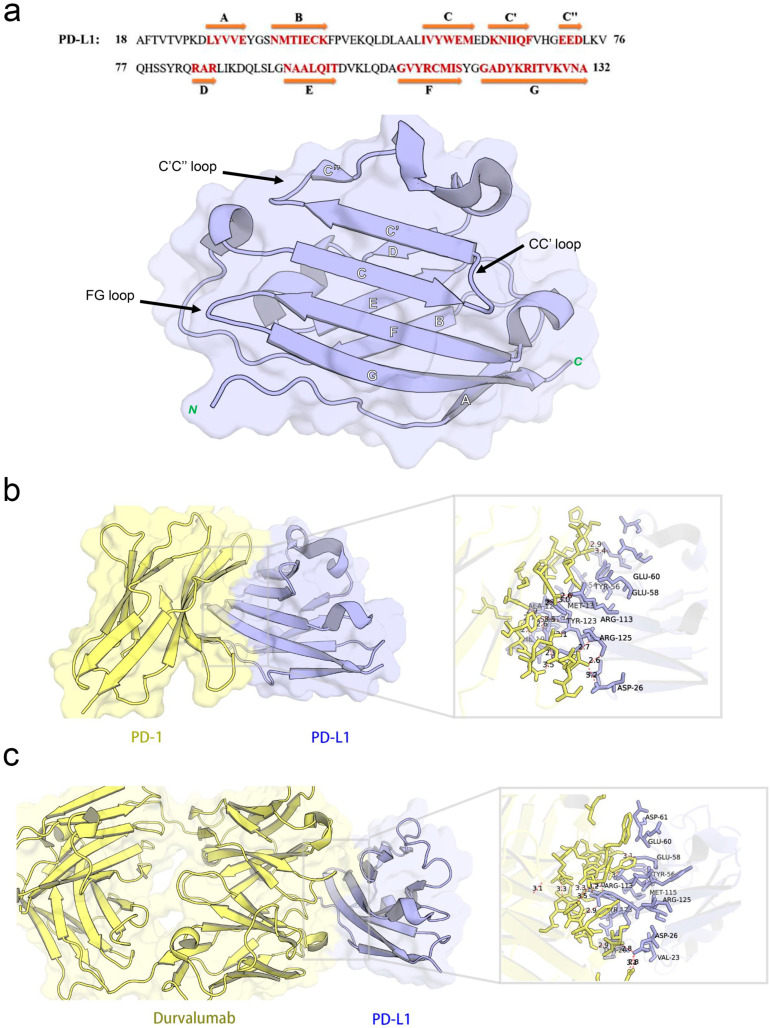
Structural characterization and interaction analyses: (**a**) crystal structures and sequence alignment of human PD-L1 protein, arrows denote eight β-strands, (**b**) binding interface between PD-1 and PD-L1, and (**c**) PD-L1 binding interface interacting with durvalumab.

The co-crystal structure of human PD-1 bound to PD-L1 (PDB ID: 4ZQK) shows that the interaction occurs at the front β-sheet face of the PD-L1 IgV domain, involving the CC’ and FG loop regions (Figure 3a). PD-L1 forms a relatively flat, hydrophobic interface (approximately 1970 Å^2^) with PD-1.

Notably, PD-L1 residues Ile54, Tyr56, Met115, Ala121, and Tyr123 interact with PD-1 residues Val64, Ile126, Leu128, Ala132, and Ile134, respectively, contributing to a hydrophobic region. In addition to hydrophobic contacts, several polar interactions stabilize the complex. Table 2 summarized the key residue contacts: for example, PD-1 Thr76 and Glu136 form hydrogen bonds and an alkyl–π interaction with PD-L1 Tyr123; PD-1 Asn66 hydrogen-bonds to PD-L1 Ala121; PD-1 Glu136 forms a salt bridge with PD-L1 Arg125, and so on. We verified these contacts by docking PD-1 alone to PD-L1 (monomer) in MOE. The docking recapitulated the same binding site and highlighted PD-L1 residues Phe19, Asp26, Ile54, Tyr56, Glu58, Glu60, Arg113, Met115, Ala121, Asp122, Tyr123, and Arg125 as directly interacting with PD-1 (Figure 3b). This provided a set of PD-L1 surface residues likely important for any molecule to block PD-1 binding.

#### 2.3.2. Binding Interface Between PD-L1 and Durvalumab

Durvalumab is an anti-PD-L1 antibody that sterically blocks PD-1 binding. The crystal structure (PDB ID: 5 × 8 M) and our PD-L1-durvalumab docking reveal that durvalumab targeted a similar region on PD-L1, centered on the CC’FG face (Figure 3c). Durvalumab interacted with 16 residues on PD-L1, including key ones it shared with PD-1 (Tyr56, Glu58, Arg113, Met115, Tyr123, and Arg125) as well as additional residues on the CC’ loop and N-terminal region (Thr20, Val23, Asp26, Glu60, and Asp61) not heavily used by PD-1. Many of these contacts are hydrogen bonds, salt bridges, or hydrophobic interactions. Our docking of durvalumab to PD-L1 confirmed that the antibody covered the PD-1 binding site on PD-L1 and also engages Asp26, Thr20, Val23, Glu60, and Asp61 via its extended loops (Figure 3c). This suggested that small molecules might achieve stronger binding if they also exploit these additional sites beyond the flat PD-1 interface.

#### 2.3.3. The Binding Patterns Between PD-L1 and Small-Molecule Inhibitors

**Figure 4:**
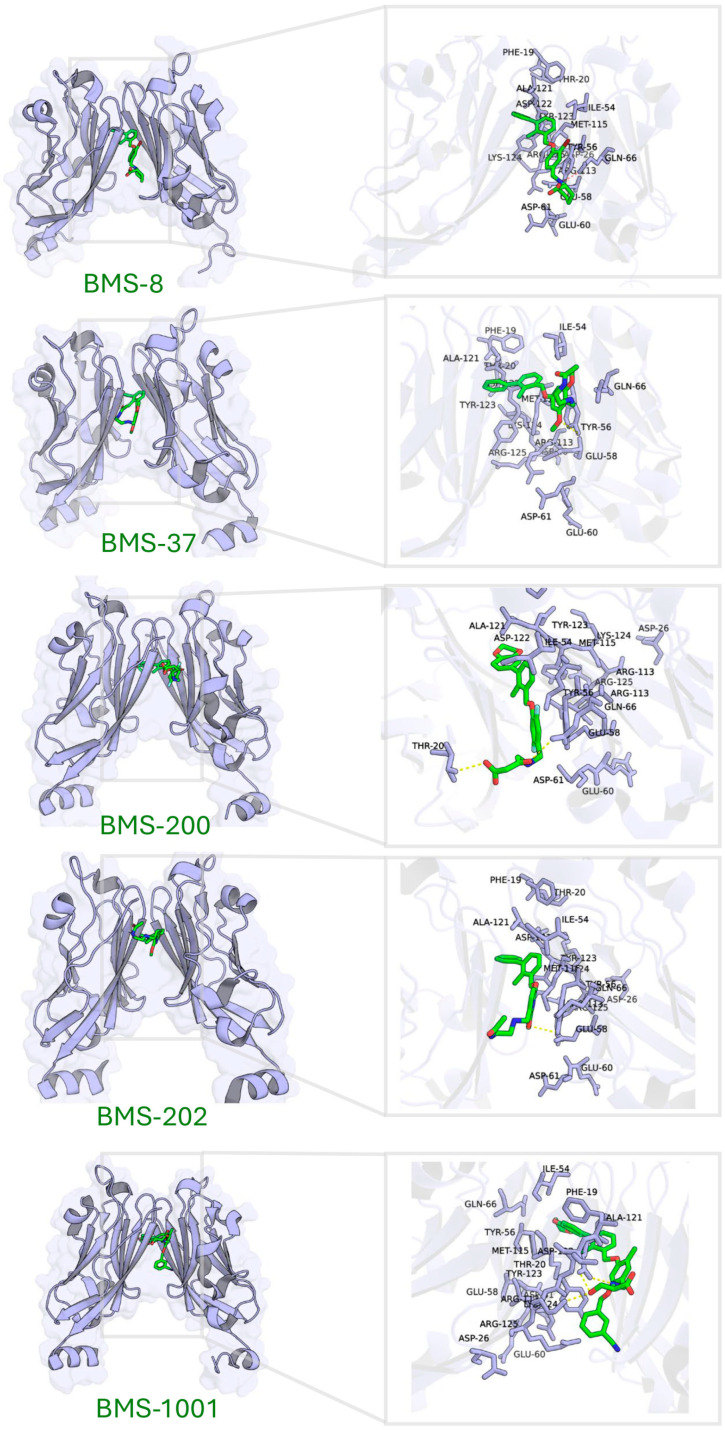
Binding site characterization of PD-L1 interacting with five small-molecule inhibitors.

Five known small-molecule inhibitors (BMS-8, BMS-202, BMS-200, BMS-37, and BMS-1001), characterized by a biphenyl core, induce PD-L1 to form homodimers in the crystal. The small molecule inserts into a hydrophobic tunnel at the PD-L1 dimer interface between the two PD-L1 protomers. As a result, this binding could simultaneously contact both protomers and subsequently block the PD-1 site (Figure 4). Despite differences in their substituents, these inhibitors display common binding features on PD-L1, as summarized in Table 3 (here, PD-L1A and PD-L1B denote the two chains of the PD-L1 dimer). Specifically, the biphenyl scaffold from small-molecule inhibitors engages PD-L1 Tyr56 (chain A) via π–π stacking, and its two phenyl rings make hydrophobic contacts with Met115 and Ala121 on both chain A and chain B of PD-L1 (π–alkyl and hydrophobic interactions). Various polar substituents on these molecules form hydrogen bonds to Asp122, Lys124, and Tyr123 (chain A), often mediated by conserved water molecules. For example, the methoxypyridine group of BMS-202 interacts with Tyr56 (chain B), Ala121 (A), Asp122 (A), and Phe19 (A) through a combination of π–π, carbonyl–π, anion–π, and water-mediated interactions. An acetamide moiety present in some analogues (BMS-202 and BMS-37) directly hydrogen-bonds to PD-L1 Lys124 (A).

Molecular docking of these inhibitors against dimeric PD-L1 confirmed consistent interaction patterns, particularly involving residues Thr20, Tyr56, Gln66, Met115, Ala121, Asp122, Tyr123, and Lys124 (Figure 4).

In summary, by integrating the structural data from PD-1, durvalumab, and small-molecule complexes, we identified 16 hotspot residues on PD-L1 that are frequently involved in binding: Phe19, Thr20, Asp26, Ile54, Tyr56, Glu58, Glu60, Asp61, Gln66, Arg113, Met115, Ala121, Asp122, Tyr123, Lys124, and Arg125. These residues, along with their prevalent interaction types (aromatic stacking, hydrophobic contacts, hydrogen bonds, and salt bridges), defined a target site for focused docking. Effective PD-L1 inhibitors are hypothesized to engage multiple hotspot residues, thereby achieving potent affinity and effectively mimicking the functional blockade provided by PD-1 or durvalumab.

#### 2.3.4. Molecular Docking-Based Virtual Screening Targeting PD-L1: Blind Docking and Hotspots Docking

We carried out molecular docking in several stages to narrow down the 460 pharmacophore-derived hits. First, a blind docking was performed using the PD-L1 monomer structure (with an unbiased search across the entire protein surface). This rapid screen, conducted in MOE with a standard scoring function, identified 247 compounds with a predicted binding free energy better than −7.5 kcal/mol. These 247 hits were considered to have at least moderate affinity for PD-L1 and were taken to the next stage. Although blind docking lacks pocket specificity and does not directly reflect functional binding affinity, it served as a practical enrichment strategy to discard compounds with minimal surface binding potential. However, we acknowledge that this filtering step might not be strictly necessary, as the entire candidate set remains computationally manageable for focused docking alone.

Next, we performed a focused docking targeting the defined PD-L1 hotspot region. In practice, this involved docking the 247 compounds to the PD-L1 monomer but emphasizing interactions with the 16 hotspot residues (Phe19, Thr20, Asp26, Ile54, Tyr56, Glu58, Glu60, Asp61, Gln66, Arg113, Met115, Ala121, Asp122, Tyr123, Lys124, and Arg125). We then filtered the results to prioritize compounds that not only had good scores but also engaged multiple hotspot residues. Specifically, we selected 33 compounds that ranked in the top 50% by docking score and formed interactions with at least three or more different PD-L1 hotspot residues. These 33 hits included 15 natural compounds (from the TCM subset) and 18 approved drugs, reflecting a mix of novel and repurposed candidates.

Finally, recognizing that the known inhibitors bind PD-L1 as dimers, we evaluated the 33 hits in the context of the PD-L1 dimer. We docked each compound into the cavity of a PD-L1 homodimer, which was extracted from the BMS-202 co-crystal structure, to see if it could occupy the hydrophobic tunnel overlapping the PD-1 binding site. We applied stringent criteria to select the most promising candidates: (1) docking energy < −7.5 kcal/mol (to ensure sufficient affinity), (2) interactions with ≥5 PD-L1 residues (spread across both protomers if possible), and (3) a binding mode that clearly occupies the hydrophobic pocket and overlaps the PD-1 interface on the dimer. After this filtering, two compounds stood out as meeting all criteria: tannic acid and anidulafungin. Tannic acid is a polyphenol (a tannin) commonly found in plants and herbs, while anidulafungin is an FDA-approved antifungal echinocandin drug. Both are relatively large molecules with multiple potential interaction points, which likely contributed to their ability to satisfy the docking requirements. These two were chosen for experimental characterization as candidate small-molecule PD-L1 inhibitors.

### 2.4. Interaction Analysis of Lead Compounds

**Figure 5:**
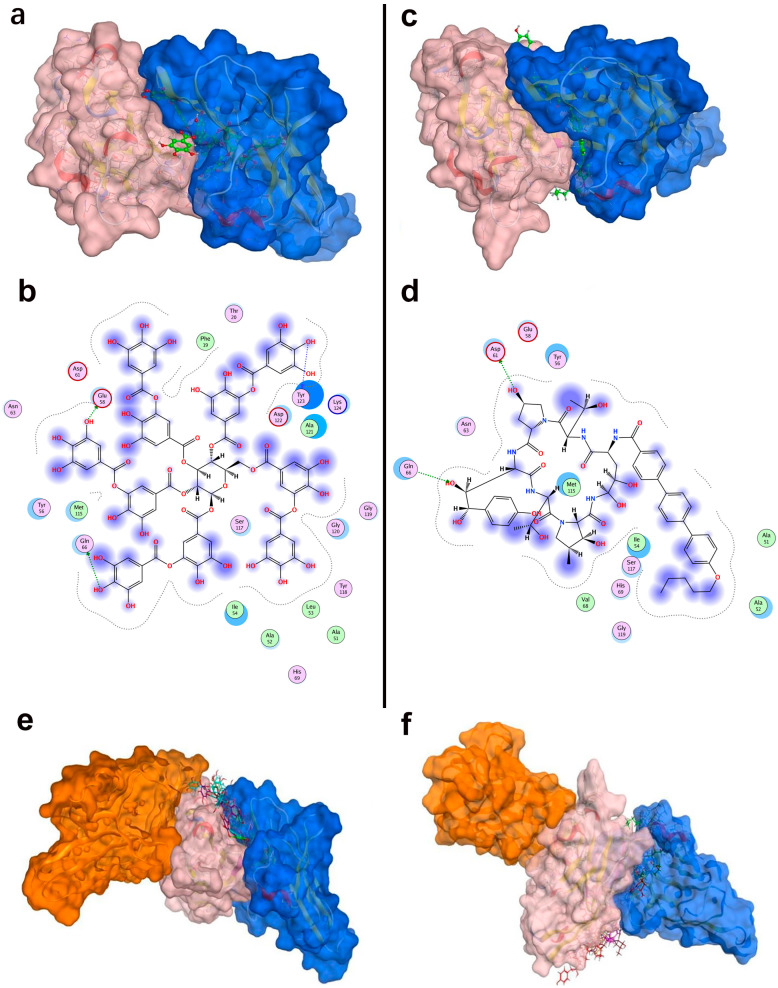
Structural interactions and conformational analysis of candidate inhibitors with PD-L1. Conformation and detailed interactions of tannic acid (**a**,**b**) and anidulafungin (**c**,**d**) bound to PD-L1; pink denotes PD-L1, and blue denotes PD-1. Docking poses illustrating PD-1 interactions with PD-L1 in the presence of tannic acid (**e**) and anidulafungin (**f**). Pink indicates PD-L1, blue denotes the PD-1 conformation without candidate compounds, and orange shows PD-1 optimal binding conformation with candidate compounds present.

We analyzed the PD-L1 binding modes of tannic acid and anidulafungin to understand how they engage the target. Table 4 summarized their key interactions with PD-L1, and Figure 5b,d show the binding poses. In the top-scoring docked pose, tannic acid fitted within the PD-L1 dimer interface, orienting several of its galloyl groups toward the protein. It formed multiple hydrogen bonds with PD-L1, notably with Glu58, Asp61, Lys75, Gln66, and Asp122. These interactions spanned both chains of the PD-L1 dimer, indicating that tannic acid simultaneously contacted different parts of the pocket. Tannic acid’s planar aromatic regions also stacked against hydrophobic patches, but its binding was dominated by polar contacts given its many hydroxyls. The predicted binding free energy for tannic acid was −9.48 kcal/mol, suggesting a relatively strong binding affinity and consistent with its extensive hydrogen-bonding network. Anidulafungin is a cyclic lipopeptide with distinct hydrophobic and hydrophilic regions. In its docked pose, anidulafungin also resided deep in the PD-L1 pocket. The lipophilic part of anidulafungin nestled into the hydrophobic cavity, where it made π-type contacts with Phe19 and Ala121 of PD-L1. Concurrently, polar groups on anidulafungin form hydrogen bonds to Asp61 and Arg113 of PD-L1. Notably, Asp61 is a residue on PD-L1’s CC’ loop that was highlighted as a hotspot that is engaged by durvalumab and tannic acid as well, and Arg113 is on the FG loop near the dimer interface. The calculated binding free energy for anidulafungin was −8.46 kcal/mol, indicating a strong binding propensity, though slightly less than tannic acid’s score.

As shown in Figure 5a,c, both tannic acid and anidulafungin clearly occupied the PD-1 binding site on PD-L1, overlapping the region where PD-1’s CC’ and FG loops would normally dock. They also penetrated deeply into the tunnel, potentially stabilizing a PD-L1 dimer-like conformation that the known biphenyl inhibitors have. This structural analysis supported the hypothesis that these molecules could function by inducing PD-L1 dimerization and blocking PD-1 access.

### 2.5. Disruption of PD-1/PD-L1 Interaction by Lead Compounds

As a stringent test of whether tannic acid and anidulafungin can prevent PD-1 from binding PD-L1, we performed a competitive docking simulation. We docked PD-1 onto the PD-L1 dimer in the presence of each compound and compared the PD-1 binding affinity and orientation with the native situation.

For the native PD-1/PD-L1 pair, the binding energy was approximately −63.9 kcal/mol, which reflected the strong protein–protein interaction. When tannic acid was bound to PD-L1, the best PD-1 docking pose had a much weaker binding energy of −45.2 kcal/mol. Similarly, with anidulafungin present, PD-1’s binding energy was −36.8 kcal/mol. Thus, both compounds dramatically reduced the PD-1 binding affinity, by approximately 20–30 kcal/mol. This reduction implied that PD-1 would bind far less tightly if these molecules occupied the interface.

Furthermore, the location of PD-1 binding shifted in these simulations. In the native complex, PD-1 aligns with PD-L1’s CC’FG face (Figure 5e,f). However, with tannic acid or anidulafungin in place, PD-1 was forced to dock at a different surface (Figure 5e,f, orange PD-1) that did not overlap the original interface. The presence of the compounds essentially blocked PD-1’s usual docking site, and the docking algorithm found an alternate, suboptimal site for PD-1 to bind. This result strongly indicated that tannic acid and anidulafungin could effectively disrupt the PD-1/PD-L1 interaction by occupying the key contact region on PD-L1.

### 2.6. In Vitro Tumor Cytotoxicity Analysis of Candidate Compounds

We first examined whether tannic acid or anidulafungin could inhibit cancer cell growth in vitro, as a preliminary indication of anti-tumor effect. Human lung adenocarcinoma A549 cells and mouse Lewis lung carcinoma (LLC) cells were treated with each compound, and cell viability was measured by the CCK-8 assay. 5-Fluorouracil (5-FU), a cytotoxic chemotherapy agent, was included as a positive control for comparison.

**Figure 6:**
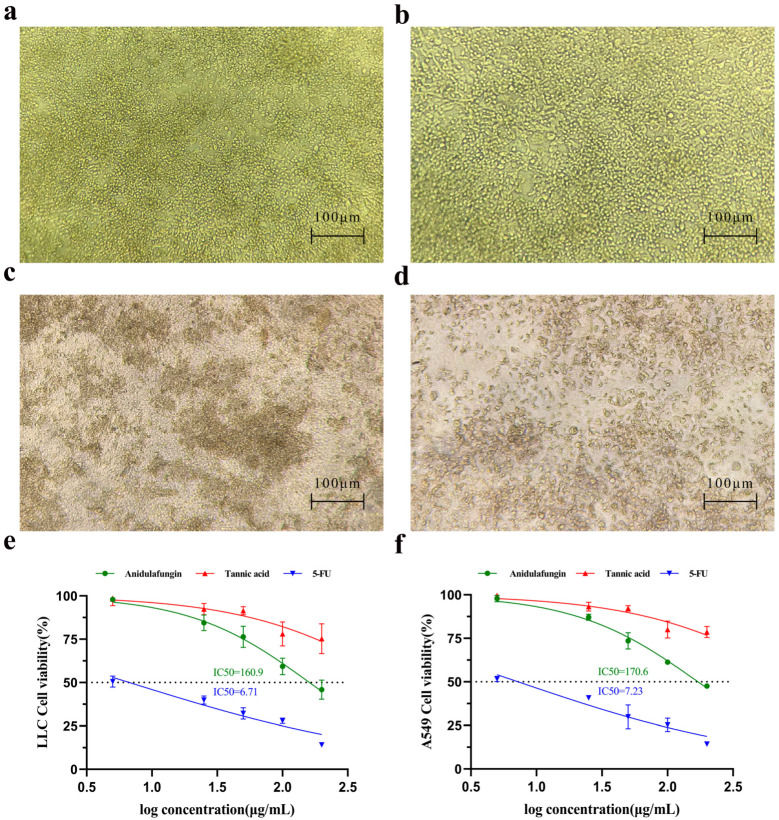
Assessment of cell growth inhibition. Initial morphology of LLC and A549 cells. (**a**–**d**) Effects of compounds on LLC cell morphology. Representative images are shown for (**a**) the negative control group, (**b**) LLC cells treated with 200 μg/mL tannic acid, (**c**) LLC cells treated with 200 μg/mL anidulafungin, and (**d**) LLC cells treated with 200 μg/mL 5-FU. (**e**,**f**) Quantitative analysis of cell viability. Dose–response curves are shown for (**e**) A549 cells and (**f**) LLC cells after treatment.

Microscopic observation provided qualitative evidence of cytotoxicity. LLC cells treated with culture medium formed irregularly shaped clusters (Figure 6a), indicating healthy proliferative cultures. In cultures treated with 200 µg/mL tannic acid, for instance, the LLC cell morphology remained similar to the untreated state (Figure 6b), with cells appearing viable and adherent and virtually no dead cells visible. In contrast, cultures treated with 200 µg/mL anidulafungin or 200 µg/mL 5-FU showed many cells rounding up or appearing as dark, shrunken bodies (Figure 6c,d). This morphology was characteristic of cell damage or death, suggesting that both anidulafungin and 5-FU caused significant cytotoxic effects, whereas tannic acid at this concentration did not.

The CCK-8 viability assays confirmed these observations quantitatively. Cell viability declined in a dose-dependent manner for all treatment groups, but the magnitude of effect differed. 5-FU was the most potent, yielding IC50 values of 6.71 µg/mL for LLC (Figure 6e) and 7.23 µg/mL for A549 (Figure 6f), respectively. Anidulafungin exhibited a moderate inhibitory effect on cell proliferation. As a result, its IC50 was 160.9 µg/mL in LLC cells and 170.6 µg/mL in A549 cells (Figure 6e,f). In contrast, tannic acid did not reach 50% growth inhibition at the tested concentrations, as cell viability in the tannic acid group remained high across doses. Therefore, anidulafungin outperformed tannic acid in these assays, justifying its selection for further study.

### 2.7. In Vitro Interaction Assay of Anidulafungin with PD-L1

**Figure 7:**
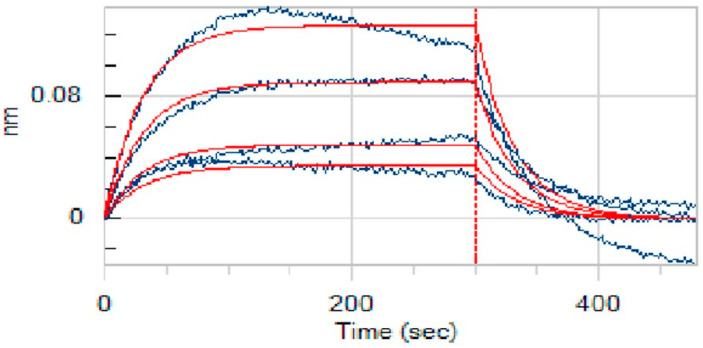
Binding affinity measurement (K_D_ values) of anidulafungin toward PD-L1. The raw experimental data, showing the binding response in nanometers (nm) over time, are represented by the blue curves. Each curve corresponds to a different concentration of anidulafungin (100, 50, 25, 12.5, and 6.25 µM). The red lines represent the best-fit curves from a 1:1 global kinetic binding model.

A key aspect of our study was to verify that anidulafungin directly binds to PD-L1, as predicted. For this, we employed bio-layer interferometry (BLI) [25], a label-free technique to measure biomolecular interactions in real time. We immobilized recombinant human PD-L1 protein on biosensor tips and then exposed them to anidulafungin at various concentrations to monitor binding. As a result, anidulafungin exhibited clear, concentration-dependent association and dissociation phases (Figure 7). Fitting the sensorgrams to a 1:1 binding model yielded an equilibrium dissociation constant (K_D_) of 76.9 µM. The kinetic rate constants were Kon = 4.08 × 10^2^ M^−1^·s^−1^ and Koff = 3.14 × 10^−2^ s^−1^, indicating a relatively fast binding and unbinding process. This measured affinity aligned well with the docking predictions and the IC_50_ range observed in cell assays, supporting that anidulafungin indeed physically interacted with PD-L1. This result indicated that anidulafungin could function as an immune checkpoint inhibitor by blocking PD-L1 on tumor cells or antigen-presenting cells, thereby promoting T-cell activation.

### 2.8. In Vivo Antitumor Activity of Anidulafungin

**Figure 8:**
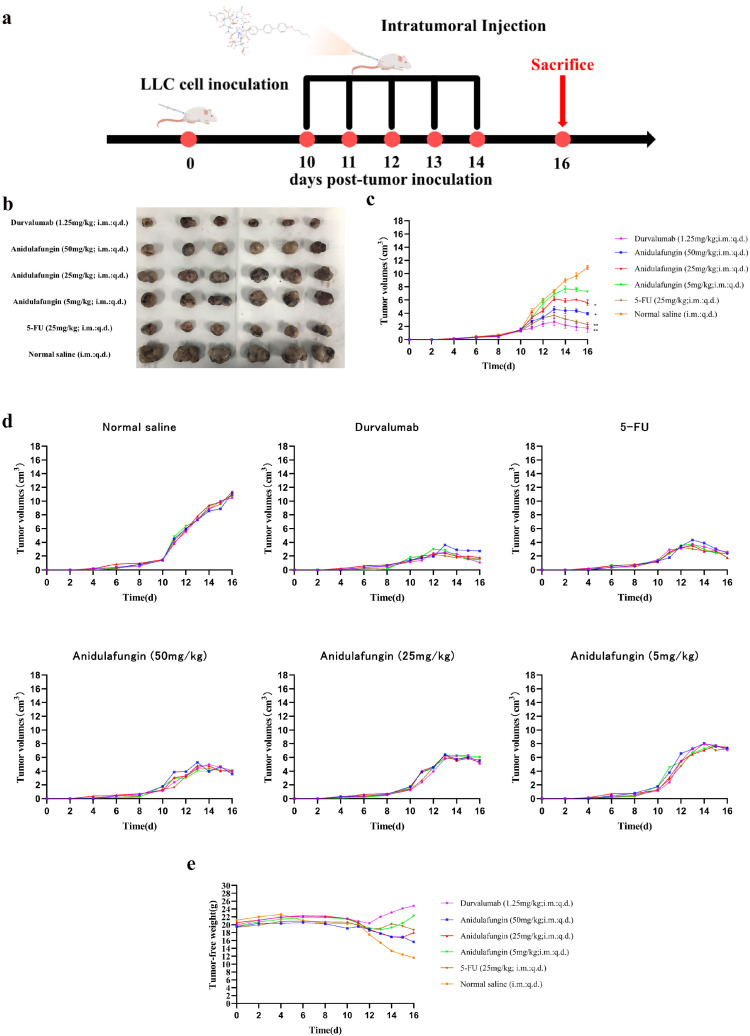
Tumor growth evaluation in vivo: (**a**) schematic illustration of tumor inoculation, drug administration, tumor volume monitoring, and analysis; (**b**) images of tumors in mice on day 16; (**c**) tumor volume changes expressed as mean ± SD (*n* = 5 per group), statistical significance: * *p* < 0.05, ** *p* < 0.01 compared to saline control; (**d**) individual tumor volumes for mice in all experimental groups; and (**e**) tumor-free body weight changes in mice.

The development of PD-1/PD-L1 small-molecule inhibitors has spanned nearly a decade, yet no drug has been approved for clinical use. In this context, repurposing anidulafungin, a drug currently used to treat serious fungal infections caused by Candida species and Aspergillus species, as a PD-L1 inhibitor appears more appealing [26]. To investigate its efficacy in treating tumors, the LLC tumor-bearing mice model was established to clarify the immune-mediated anti-tumor effects of anidulafungin in vivo. We tested the inhibitory effects of different concentrations of anidulafungin, durvalumab, and 5-FU on tumor growth (Figure 8a). We established LLC tumors in mice and then treated them with anidulafungin at three dose levels (5, 25, and 50 mg/kg) via intratumoral injection once daily for 5 days. Although anidulafungin is normally given intravenously for fungal infections, intratumoral administration in this study was chosen to maximize local drug exposure, and we wanted to ensure it reached the tumor. For comparison, groups of tumor-bearing mice received either durvalumab (1.25 mg/kg intratumorally, as a positive control PD-L1 blockade therapy), 5-FU (25 mg/kg, a chemotherapy positive control), or saline (vehicle control). Tumor volumes were monitored over the treatment period. We also evaluated their impact on the plasma levels of IFN-γ and IL-4, as well as their effects on the expression of IFN-γ and granzyme B (GZMB) in the tumor tissue.

As shown in Figure 8c,d, tumors grew rapidly in mice receiving saline. In contrast, the anidulafungin medium-dose (25 mg/kg) group, the anidulafungin high-dose (50 mg/kg) group, the 5-FU group, and the durvalumab group remarkably inhibited tumor growth. The anidulafungin low-dose (5 mg/kg) group, anidulafungin medium-dose (25 mg/kg) group, anidulafungin high-dose (50 mg/kg) group, 5-FU group, and durvalumab group resulted in tumor growth inhibitions (TGIs) of 24.77%, 45.34%, 63.89%, 78.13%, and 89.17%, respectively (Table 5). The efficacy of high-dose anidulafungin (50 mg/kg) was comparable to durvalumab. This suggested that anidulafungin has a significant inhibitory effect on tumor growth. This is impressive considering durvalumab is a high-affinity antibody and anidulafungin, at this point, is a relatively unoptimized small molecule. The data suggested that, when delivered at a sufficient dose and concentration in the tumor, anidulafungin could elicit an anti-tumor effect on par with an established immunotherapeutic [26,27].

Notably, we observed no overt toxicity from anidulafungin in the treated mice. All groups receiving treatment (anidulafungin, 5-FU, and durvalumab) had higher tumor-free body weights (body weight minus tumor weight) than the saline group. In particular, 5-FU and high-dose anidulafungin maintained body weight well, indicating that tumor reduction was not accompanied by severe systemic illness or weight loss (Figure 8e). This suggested that anidulafungin was relatively well-tolerated at the doses used, and unlike 5-FU, its mechanism is not simply general cytotoxicity, which often causes weight loss.

### 2.9. Immune Response Analysis

To verify that anidulafungin’s anti-tumor effect was indeed immune-mediated, we examined immune markers in the mice. Specifically, we measured Th1 cytokine IFN-γ and Th2 cytokine IL-4 levels in serum, as these cytokines can reflect an immune response against tumors (Table 6). Additionally, we assessed intratumoral expression of IFN-γ and granzyme B (GZMB) via immunohistochemistry, as these are hallmarks of an active cytotoxic T-cell response within the tumor.

**Figure 9:**
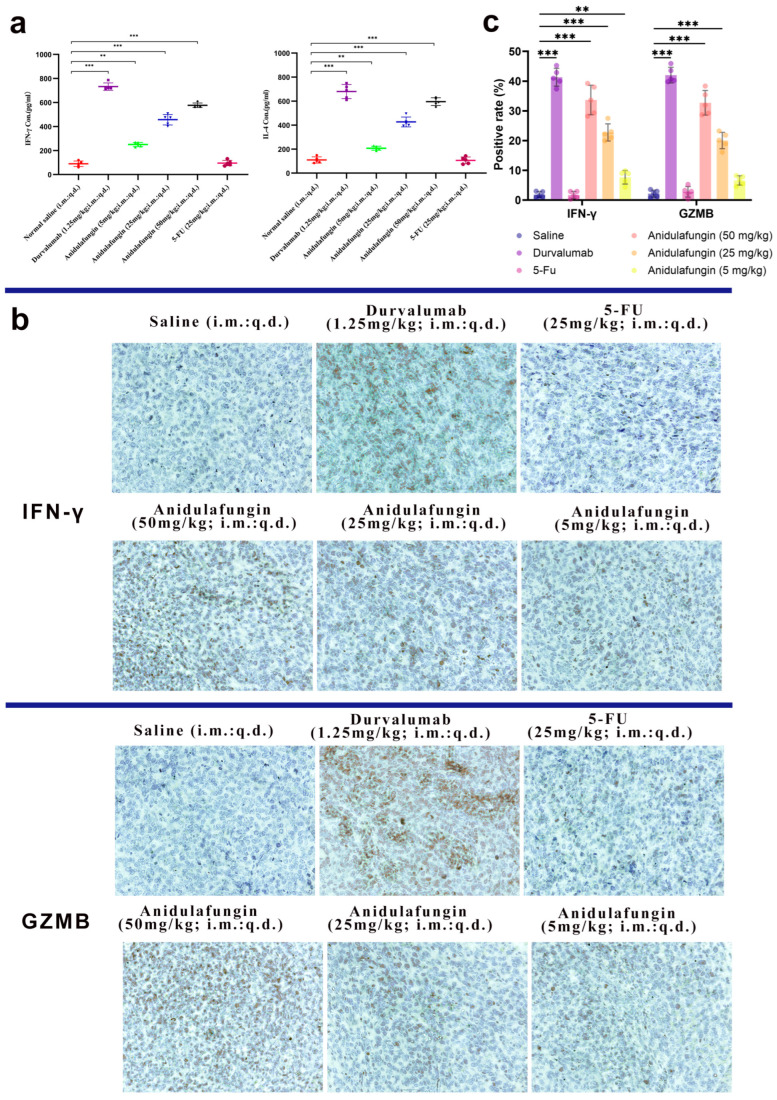
Immunological response assessment: (**a**) serum IFN-γ and IL-4 levels (*n* = 5), statistical significance indicated by ** *p* < 0.01 and *** *p* < 0.001; (**b**) representative images showing IFN-γ and GZMB expression in tumor tissues, 200× magnification; and (**c**) quantitative analysis of positive rates for IFN-γ and GZMB expression; ** *p* < 0.01 and *** *p* < 0.001 indicate significant differences between the saline control group and the anidulafungin medium-dose (25 mg/kg), anidulafungin high-dose (50 mg/kg), or durvalumab treatment groups.

Blood was collected 24 h after the final treatment and analyzed by ELISA for IFN-γ and IL-4 (Figure 9a). In the anidulafungin-treated groups, the levels of both cytokines were elevated compared to the saline group, and this elevation was dose-dependent. For instance, mice treated with medium- or high-dose anidulafungin showed significantly higher IFN-γ and IL-4 concentrations in serum than the control mice (Table 6, *p* < 0.05). The levels in the high-dose group approached those in the durvalumab group, which showed the highest cytokine induction as expected. However, mice treated with 5-FU had IFN-γ and IL-4 levels similar to controls, consistent with chemotherapy’s mode of action not primarily involving immune activation. These results indicated that anidulafungin triggered a systemic immune response, skewing towards both Th1 (IFN-γ) and Th2 (IL-4) cytokine production. The concurrent rise in IFN-γ and IL-4 upon PD-L1 blockade was in line with literature reports for PD-1/PD-L1 checkpoint inhibitors, which often enhanced both cytotoxic T cell (IFN-γ) and helper T cell (IL-4) responses. The fact that anidulafungin, but not 5-FU, increased these cytokines revealed that anidulafungin’s anti-tumor effect likely involved immune system engagement rather than direct killing of tumor cells.

We next looked at the tumors themselves for evidence of T-cell-mediated activity. IFN-γ is mainly produced by activated T cells and NK cells, and GZMB is a cytotoxic granule enzyme typically released by CD8+ T cells and NK cells to induce apoptosis in target cells. Tumor sections were stained for these markers. The durvalumab-treated tumors had large areas of brown staining for IFN-γ and GZMB, reflecting a robust immune cell infiltration and activation in those tumors (Figure 9b,c). Similarly, anidulafungin (25 and 50 mg/kg) tumors showed extensive IFN-γ and GZMB positivity, though slightly less uniformly than durvalumab. Even the low-dose anidulafungin group (5 mg/kg) exhibited more IFN-γ/GZMB-positive cells than the saline control, albeit in smaller patches. In contrast, 5-FU-treated tumors had very little IFN-γ or GZMB staining, appearing similar to the saline group with only sparse positive cells. These qualitative observations confirmed that anidulafungin activated immune effector mechanisms inside the tumor, akin to PD-L1 antibody therapy, whereas 5-FU did not recruit immune effectors. For a quantitative perspective, we calculated the percentage of IFN-γ+ and GZMB+ cells in each tumor (Table 7). In saline-treated tumors, these percentages were near 2%. Durvalumab raised IFN-γ+ to approximately 41% and GZMB+ to 42%, representing a massive immune infiltration/activation. Anidulafungin at 25 mg/kg achieved 23% IFN-γ+ and ~20% GZMB+, and at 50 mg/kg achieved 34% IFN-γ+ and 33% GZMB+. All these values were significantly higher than the control (*p* < 0.05). Low-dose anidulafungin and 5-FU groups did not show statistically significant increases. The slight increase in low-dose anidulafungin still hinted that even a small amount of PD-L1 blockade can begin to activate T cells.

Overall, the immunohistochemistry results supported that anidulafungin’s tumor inhibition was immune-mediated. The high-dose anidulafungin effect was roughly 75–80% as efficacious as durvalumab in terms of inducing IFN-γ and GZMB in the tumor. This was remarkable for a small molecule and suggested that anidulafungin engaged the adaptive immune system to attack the tumor, functioning in a similar manner to a checkpoint inhibitor antibody. Unlike traditional chemotherapy 5-FU, anidulafungin reinvigorated T cells to kill the cancer. This mechanism is much more specific and can lead to immunological memory against the tumor.

In summary, the in vivo experiments demonstrated that anidulafungin is effective in suppressing tumor growth through an immune-driven mechanism. The cytokine release and T-cell activation patterns mirrored those of known PD-L1/PD-1 blockade therapies, confirming that our virtual screening successfully identified a compound that could mimic the pharmacological action of PD-L1 antibodies. These results positioned anidulafungin as a promising immunotherapeutic agent. As a repurposed drug, it offers the advantage of known safety and pharmacology, which could accelerate its development timeline for oncology indications. Future optimizations, including analog development to improve potency or formulation changes to enhance tumor delivery, could further increase its efficacy. This study also demonstrated the power of combining ligand-based and structure-based computational methods in drug discovery. By using pharmacophore modeling to capture the essential features of PD-L1 ligands and docking to refine hits against detailed protein interaction hotspots, we successfully navigated a large chemical space to discover functional hits. The approach described here can serve as a general framework for discovering new small-molecule agents targeting other protein–protein interactions in immuno-oncology. Future efforts will focus on optimizing anidulafungin’s chemical structure for enhanced potency and assessing its efficacy and safety in additional tumor models. Ultimately, our findings open a new avenue for small-molecule immune checkpoint inhibitors, with anidulafungin as a promising lead for further development in cancer therapy.

## 3. Materials and Methods

### 3.1. Materials and Reagents

Human lung cancer A549 cells and mouse Lewis lung carcinoma (LLC) tumor cells were purchased from the Type Culture Collection of the Chinese Academy of Sciences (Shanghai, China). Tannic acid (CAS No. 18483-17-5) and fluorouracil (5-FU) were purchased from Bidepharm (Shanghai, China). Anidulafungin (CAS No. 166663-25-8) was obtained from Tianjin Derchemist Sci-Tech Co., Ltd. (Tianjin, China). DMEM, fetal bovine serum (FBS), and penicillin-streptomycin were obtained from Sigma-Aldrich (Darmstadt, Germany). All the mouse ELISA kits were purchased from Sino Biological Inc., Beijing, China. Formalin fixative solution, xylene, and dimethyl sulfoxide (DMSO) were purchased from Sinopharm Chemical Reagent Co. (Shanghai, China).

### 3.2. Preparation and Preprocessing of Training and Test Sets, and Screening Dataset

A series of small molecules capable of inhibiting the interaction between PD-1 and PD-L1 was collected from PubMed and the China National Intellectual Property Administration (CNIPA). These compounds included biphenyl derivatives, heteroaryl-substituted biphenyl derivatives, dithiazole and thiazole derivatives, benzyl phenyl ether derivatives, as well as imidazo[4,5-c]pyridine and other heterocyclic compounds, with IC50 values ranging from 10^−4^ to 10^2^ nM. Additionally, the crystal structures of five known small-molecule inhibitors in complex with PD-L1 were collected from the RCSB Protein Data Bank (PDB): BMS-8 (PDB ID: 5J8O), BMS-202 (PDB ID: 5J89), BMS-200 (PDB ID: 5N2F), BMS-37 (PDB ID: 5N2D), and BMS-1001 (PDB ID: 5NIU). A total of 3906 compounds were assembled into a screening dataset. This dataset included 1876 small molecules derived from 90 immunomodulatory and anti-tumor traditional Chinese medicines (TCMs), obtained from the Traditional Chinese Medicine Systems Pharmacology (TCMSP) and PubChem databases, and 2030 approved small-molecule drugs from TargetMol. Molecular Operating Environment (MOE) 2022 was used to minimize the energy of 62 small molecules for pharmacophore model construction, along with the 3906 candidate compounds for virtual screening.

### 3.3. Generation of Pharmacophore Models

The prepared training dataset of compounds was imported into MOE 2022. A conformation search was performed to generate all possible conformations for each compound. In the Pharmacophore Elucidate module, the activity value was set to 0.9, and the number of pharmacophore features was set to 4–5, with other parameters maintained at their default values. After molecular superimposition, five pharmacophore models (Hypo1 to Hypo5) were generated (Figure 2). The pharmacophoric features included hydrogen bond acceptor (A), hydrogen bond donor (D), hydrophobic feature (H), and ring aromatic feature (R). Among these, three pharmacophore models (Hypo1, Hypo2, and Hypo3) contained five pharmacophore features, while the remaining models (Hypo4 and Hypo5) contained four features each (Figure 2).

### 3.4. Docking Procedure

MOE 2022 was used to extract dimeric PD-L1 from the crystal structure of known small-molecule inhibitors in complex with PD-L1. Additionally, single PD-L1 and PD-1 chains, as well as durvalumab, were extracted from the PD-1/PD-L1 complexes (PDB ID: 4ZQK) and PD-L1/durvalumab complexes (PDB ID: 5X8M). Crystallographic water molecules were removed from all extracted structures, defective atoms were fixed, and the structures were energy-minimized.

To identify hotspot residues within the PD-L1 binding pocket, molecular docking was employed to visualize the binding interactions between PD-L1 and PD-1, between PD-L1 and durvalumab, and between PD-L1 and five known PD-L1 inhibitors. To validate the binding site identified from the crystal structures, PD-1 and durvalumab were docked with PD-L1, and the five known inhibitors were docked with dimeric PD-L1 using MOE.

### 3.5. Cytotoxicity Assay

Human lung cancer A549 cells and mouse Lewis lung carcinoma (LLC) tumor cells were purchased from the Type Culture Collection of the Chinese Academy of Sciences (Shanghai, China).

Tannic acid, anidulafungin, and 5-FU were dissolved in DMSO to prepare 100 mg/mL stock solutions. A complete Dulbecco’s Modified Eagle Medium (DMEM; Hyclone, Logan, UT, USA) was supplemented with 10% (*v*/*v*) heat-inactivated FBS (Hyclone) and 1% (*v*/*v*) penicillin-streptomycin (Sijiqing, Hangzhou, China) [28]. A logarithmic dilution series (5, 25, 50, 100, and 150 μg/mL) was prepared in pre-warmed medium (37 °C). A549 and LLC cells were seeded in 96-well plates with 100 μL medium per well and incubated for 24 h at 37 °C in 5% CO_2_. The medium was aspirated, and 100 μL of test compounds were added in reverse concentration order (high to low) to minimize cross-contamination. A Cell Counting Kit-8 (CCK-8) working solution (Dojindo Laboratories, Kumamoto, Japan) was prepared. Ten microliters of reagent were added per well using a multichannel pipette, ensuring that no bubbles were formed. The plates were gently shaken and incubated at 37 °C for 1–2 h. Absorbance was measured at 450 nm using a microplate reader (BioTek Instruments, Winooski, VT, USA).

### 3.6. Binding Affinity Assay

The ForteBio (Menlo Park, CA, USA) Octet RED96e bio-layer interferometry system was configured with protein buffer, protein solution, analyte buffer, and analyte solutions at varying concentrations. Protein buffer was formulated as PBS buffer containing 0.1% BSA and 0.02% Tween 20 [29]. The PD-L1 protein solution was prepared by reconstituting lyophilized PD-L1 powder in protein buffer to achieve a final concentration of 10 µg/mL. Analyte buffer was prepared by supplementing PBS buffer with 1% DMSO, 0.1% BSA, and 0.02% Tween 20. Analyte solutions were generated by dissolving anidulafungin in DMSO to prepare a 500 µM stock solution, followed by serial dilution with PBS buffer containing 0.1% BSA and 0.02% Tween 20 to obtain working concentrations of 100, 50, 25, 12.5, and 6.25 µM.

### 3.7. In Vivo Anti-Tumor Activity

All animal studies were approved by the Institutional Animal Care and Use Committee at Jiangsu University and conducted following institutional guidelines. Female SPF-grade BALB/c mice aged 6–8 weeks were purchased from Jiangsu University Experimental Animal Center (Zhenjiang, China).

A 0.2 mL aliquot of LLC cell suspension was subcutaneously injected into the axillary region of mice. Tumors exceeding 1 cm^3^ became palpable at the injection site approximately 9 days post-inoculation, achieving a 100% tumorigenesis rate, confirming successful model establishment. Mice were randomized into six groups (*n* = 5): a negative control group (normal saline), an anidulafungin low-dose group (5 mg/kg), a medium-dose group (25 mg/kg), a high-dose group (50 mg/kg), a 5-FU group (25 mg/kg), and a durvalumab group (1.25 mg/kg). Intratumoral injections were administered daily for five consecutive days using a 0.2 mL volume per dose. Needles were inserted perpendicularly at the tumor periphery (2–3 mm from the edge) toward the central region. Blood samples were collected via retro-orbital puncture 24 h post-treatment cessation, and serum IFN-γ/IL-4 levels were quantified using ELISA kits (Sino Biological Inc., Beijing, China). Excised tumors were fixed in 10% neutral-buffered formalin, paraffin-embedded, and sectioned into 7 μm thick slices. After blocking with goat serum, sections were incubated with rabbit anti-GZMB and anti-IFN-γ primary antibodies overnight at 4 °C, followed by 1-h incubation with horseradish peroxidase (HRP)-conjugated secondary antibodies at room temperature. Chromogenic development was performed using 3,3′-diaminobenzidine (DAB) for 10 min prior to microscopic analysis.

### 3.8. Statistical Analysis

All data were analyzed and graphed using GraphPad Prism 8.0. The experimental data were presented as mean ± SD of three independent trials. Statistical differences between two groups were analyzed by the two-sided Student’s *t*-test, while differences among multiple groups were analyzed by one-way ANOVA, followed by Tukey’s multiple comparisons. *p* < 0.05 was considered statistically significant.

## 4. Conclusions

In conclusion, we have identified anidulafungin as a potent small-molecule PD-L1 inhibitor through a comprehensive virtual screening and experimental validation strategy. Anidulafungin showed significant antitumor activity in vitro and in vivo, comparable in effect to a clinically approved anti-PD-L1 antibody in our models. Mechanistically, anidulafungin reactivated anti-tumor immunity by blocking the PD-1/PD-L1 interaction, thereby mimicking the immune checkpoint blockade achieved by monoclonal antibodies. As an FDA-approved antifungal drug, anidulafungin has a well-established safety profile; its development as a cancer immunotherapy could be expedited relative to a novel compound, bypassing many early-stage development hurdles.

## Figures and Tables

**Table 1:** Validation of pharmacophore models based on test set.

Pharmacophore Models	Number of Compounds	Match Count	Match Rate
Hypo1	30	28	93.3%
Hypo2	30	29	96.7%
Hypo3	30	28	93.3%
Hypo4	30	22	73.33%
Hypo5	30	24	80.00%

Note: Match count denotes the number of compounds in the test set that satisfied all features of the pharmacophore. The match rate was calculated as the proportion of test set compounds that satisfied all pharmacophore features relative to the total number of test set compounds.

**Table 2:** The interactions between PD-1 and PD-L1.

PD-1 Interacting Residues	PD-L1 Interacting Residues	Interaction Patterns
Ile134_PD-1,_ Glu136_PD-1_, and Thr76_PD-1_	Tyr123_PD-L1_	Alkyl-π hyperconjugation and hydrogen bond
Asn66_PD-1_	Ala121_PD-L1_	Hydrogen bond
Tyr68_PD-1_	Asp122_PD-L1_	Hydrogen bond
Glu136_PD-1_	Arg125_PD-L1_ and Arg113_PD-L1_	Hydrogen bond and salt bridge
Gln75_PD-1_	Arg125_PD-L1_ and Asp26_PD-L1_	Hydrogen bond
Ile134_PD-1_	Glu58_PD-L1_ and Glu60_PD-L1_	Hydrogen bond
Ala132_PD-1_	Gln66_PD-L1_	Hydrogen bond
Thr76_PD-1_	Tyr124_PD-L1_	Hydrogen bond
Lys78_PD-1_	Phe19_PD-L1_	Hydrogen bond
Asn66_PD-1_	Ala121_PD-L1_	Hydrogen bond

**Table 3:** The interactions between PD-L1 and small-molecule inhibitors.

Groups of Small-Molecule Compounds	PD-L1 Interacting Residues	Interaction Patterns
Benzene ring with biphenyl structure	Tyr56_PD-L1A_	T-Stacking (π–π stacking)
Biphenyl structural terminal benzene ring	Met115_PD-L1A_ Ala121_PD-L1B_	π–alkyl interactions
Biphenyl intermediate phenylmethyl ring	Met115_PD-L1B_ Ala121_PD-L1A_	Hydrophobic interaction
Methoxypyridine of BMS202	Tyr56_PD-L1B_ Ala121_PD-L1A_ Asp122_PD-L1A_ Phe19_PD-L1A_	π–π conjugation, carbonyl–π interactions, anion–π interactions, and water molecule-mediated lone electron pair–π interactions
BMS202 with the methoxy group of BMS37, the carboxyl and hydroxyl groups of BMS-8, BMS-200, and BMS-1001	Asp122_PD-L1A_ Lys124_PD-L1A_ Tyr123_PD-L1A_	Water molecule-mediated hydrogen bonding interactions
Acetamide of BMS202 and BMS37	Lys124_PD-L1A_	Hydrogen bonding interaction
iphenyl side chains of BMS-8, BMS200, and BMS-37 and the five-membered ring N	Gln66_PD-L1B_ Thr20_PD-L1A_	Hydrogen bonding interaction

Note: PD-L1A denotes the A chain of the dimeric PD-L1, while PD-L1B denotes the B chain of the dimeric PD-L1.

**Table 4:** The interactions between PD-L1 and candidate compounds.

Compounds	Binding Sites on Compounds	Interacting Residues of PD-L1	Interaction Patterns
Tannic acid	O-33	Ala121	H-donor
	O-41	Asp122	H-donor
	O-41, O-36, O-32	Glu58	H-donor
	C-50, O-21, O-25	Asp61	H-donor
	O-20	Lys75	H-donor
	O-27	Gln66	H-acceptor
	C-105	Tyr123	H-pi
Anidulafungin	O-97, N-59	Asp61	H-donor
	O-90, O-15, O-45	Arg113	H-acceptor
	6-ring	Phe19	pi-H
	6-ring, O-45	Ala121	pi-H, H-acceptor
	6-ring	Lys124	pi-cation
	O-84	Ser117P	H-acceptor
	6-ring	Gly120	pi-H

Note: “O-33” denotes the oxygen atom at position 33 in the compound, and “6-ring” denotes the benzene ring. “H-donor” signifies the role of PD-L1 as a hydrogen bond donor in molecular interactions, while “H-acceptor” denotes PD-L1’s role as a hydrogen bond acceptor in these interactions. “pi-H” and “H-pi” denote π-bond interactions, and “pi-cation” denotes π-cation interactions.

**Table 5:** The anti-tumor effect of treated groups (mean ± SD, *n* = 5).

Groups	Tumor Volumes (cm^3^)	Tumor Weights (g)	TGI (%)
Durvalumab (1.25 mg/kg)	1.72 ± 0.63	1.8 ± 1.01	89.17
Anidulafungin (5 mg/kg)	7.27 ± 0.24	7.5 ± 0.97	24.77
Anidulafungin (25 mg/kg)	5.59 ± 0.84	5.45 ± 0.89	45.34
Anidulafungin (50 mg/kg)	3.95 ± 0.21	3.60 ± 0.91	63.89
5-FU (25 mg/kg)	2.24 ± 0.31	2.18 ± 0.47	78.13
Normal saline	10.93 ± 0.35	9.97 ± 0.87	-

**Table 6:** Serum levels of IL-6, IL-4, and IFN-γ at the end of treatment of each group.

Groups	Normal Saline	Durvalumab (1.25 mg/kg)	Anidulafungin (5 mg/kg)	Anidulafungin (25 mg/kg)	Anidulafungin (50 mg/kg)	5-FU (25 mg/kg)
IFN-γ (pg/mL)	89.47 ± 22.96	732.17 ± 30.74	249.92 ± 17.66	456.57 ± 39.50	576.43 ± 18.99	95.35 ± 22.34
IL-4 (pg/mL)	109.42 ± 26.11	679.95 ± 59.08	207.32 ± 16.92	426.14 ± 36.75	595.42 ± 31.96	105.73 ± 28.54

**Table 7:** IFN-γ and GZMB expression in tumor tissues.

Groups	Normal Saline	Durvalumab (1.25 mg/kg)	Anidulafungin (5 mg/kg)	Anidulafungin (25 mg/kg)	Anidulafungin (50 mg/kg)	FU (25 mg/kg)
IFN-γ positive rate (%)	1.85 ± 0.97	41.30 ± 2.99	7.67 ± 2.28	22.73 ± 2.90	33.67 ± 4.96	1.72 ± 1.19
GZMB positive rate (%)	2.18 ± 1.15	42.03 ± 2.62	6.61 ± 1.57	20.03 ± 2.75	32.73 ± 4.13	2.78 ± 1.77

## Data Availability

The raw data supporting the conclusions of this article will be made available by the authors on request. The data are not publicly available due to their involvement in ongoing related research.

## Supplementary Materials

The following supporting information can be downloaded at: https://www.mdpi.com/article/10.3390/ph18081209/s1, Figure S1. Structures of 30 small molecules for test dataset.

## Abbreviations

The following abbreviations are used in this manuscript:

PD-L1	Programmed death ligand 1
PD-1	Programmed cell death protein 1
LLC	Lewis lung carcinoma
BLI	Biolayer interferometry
K_D_	Dissociation constant
GZMB	Granzyme B
CADD	Computer-Assisted Drug Design
FBS	Fetal bovine serum
DMSO	Dimethyl sulfoxide
PDB	Protein Data Bank
TCMs	Traditional Chinese medicines
TCMSP	Traditional Chinese Medicine Systems Pharmacology
DMEM	Dulbecco’s Modified Eagle Medium
CCK-8	Cell counting kit-8
CNIPA	China National Intellectual Property Administration
MOE	Molecular operating environment
