# Supplementary figures and images for: Discovery of Small-Molecule PD-L1 Inhibitors via Virtual Screening and Their Immune-Mediated Anti-Tumor Effects
Source: Pharmaceuticals (Basel). 2025 Aug 15;18(8):1209. doi: 10.3390/ph18081209 (PMC12388884; doi:10.3390/ph18081209)

# Supplementary Materials

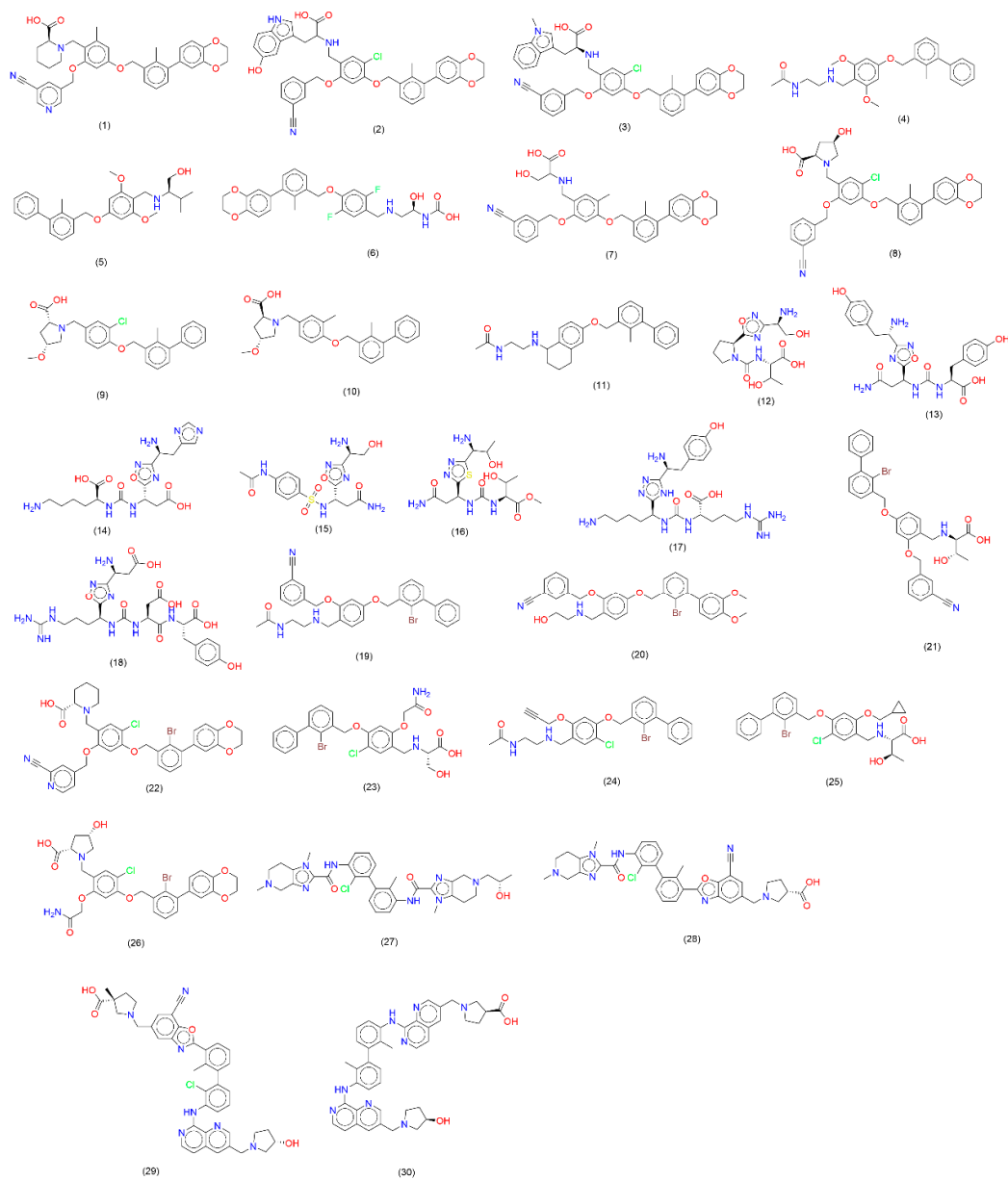

**Figure S1.** Structures of 30 small molecules for test dataset.

Supplement: Supplementary file 1 [file pharmaceuticals-18-01209-s001.zip › pharmaceuticals-3789098-supplementary.pdf]
